# Accuracy of *in vivo* microCT imaging in assessing the microstructural properties of the mouse tibia subchondral bone

**DOI:** 10.3389/fendo.2022.1016321

**Published:** 2023-01-11

**Authors:** S. Oliviero, E. Millard, Z. Chen, A. Rayson, B.C. Roberts, H.M.S. Ismail, I. Bellantuono, E. Dall’Ara

**Affiliations:** ^1^ Insigneo Institute for in silico Medicine, University of Sheffield, Sheffield, United Kingdom; ^2^ Department of Oncology and Metabolism, University of Sheffield, Sheffield, United Kingdom; ^3^ Department of Industrial Engineering, Alma Mater Studiorum, University of Bologna, Bologna, Italy; ^4^ Department of Infection, Immunity and Cardiovascular Disease, University of Sheffield, Sheffield, United Kingdom; ^5^ Healthy Lifespan Institute, University of Sheffield, Sheffield, United Kingdom

**Keywords:** osteoarthritis, mouse models, DMM, microCT, subchondral bone

## Abstract

Osteoarthritis (OA) is one of the most common musculoskeletal diseases. OA is characterized by degeneration of the articular cartilage as well as the underlying subchondral bone. Post-traumatic osteoarthritis (PTOA) is a subset of OA caused by mechanical trauma. Mouse models, such as destabilization of the medial meniscus (DMM), are useful to study PTOA. *Ex vivo* micro-Computed Tomography (microCT) imaging is the predominant technique used to scan the mouse knee in OA studies. Nevertheless, *in vivo* microCT enables the longitudinal assessment of bone microstructure, reducing measurement variability and number of animals required. The effect of image resolution in measuring subchondral bone parameters was previously evaluated only for a limited number of parameters. The aim of this study was to evaluate the ability of *in vivo* microCT imaging in measuring the microstructural properties of the mouse tibia trabecular and cortical subchondral bone, with respect to *ex vivo* high resolution imaging, in a DMM model of PTOA. Sixteen male C57BL/6J mice received DMM surgery or sham operation at 14 weeks of age (N=8 per group). The right knee of each mouse was microCT scanned *in vivo* (10.4μm voxel size) and *ex vivo* (4.35μm voxel size) at the age of 26 weeks. Each image was aligned to a reference image using rigid registration. The subchondral cortical bone plate thickness was measured at the lateral and medial condyles. Standard morphometric parameters were measured in the subchondral trabecular bone. *In vivo* microCT imaging led to significant underestimation of bone volume fraction (-14%), bone surface density (-3%) and trabecular number (-16%), whereas trabecular thickness (+3%) and separation (+5%) were significantly overestimated. Nevertheless, most trabecular parameters measured *in vivo* were well correlated with *ex vivo* measurements (R^2 =^ 0.69-0.81). Degree of anisotropy, structure model index and connectivity density were measured *in vivo* with lower accuracy. Excellent accuracy was found for cortical thickness measurements. In conclusion, this study identified what bone morphological parameters can be reliably measured by *in vivo* microCT imaging of the subchondral bone in the mouse tibia. It highlights that this approach can be used to study longitudinal effects of diseases and treatments on the subchondral cortical bone and on most subchondral trabecular bone parameters, but systematic over- or under-estimations should be considered when interpreting the results.

## 1 Introduction

Osteoarthritis (OA) is one of the most common musculoskeletal diseases, affecting more than 240 million people worldwide (1). OA is characterized by degeneration and thinning of the articular cartilage, joint inflammation, alterations of the underlying subchondral bone and formation of osteophytes, causing joint pain and reduced mobility (1). Post-traumatic osteoarthritis (PTOA) is a subset of OA caused by mechanical trauma, such as rupture of the anterior cruciate ligament and meniscal tears (2).

Mouse models of spontaneous or surgically-induced OA are important to investigate the disease mechanisms with rapid and relatively low cost studies (3). Different types of models have been developed, that include chemically-induced cartilage degeneration, surgical models and genetic models (3). Among those, surgical models are those most used to investigate PTOA as they mimic clinical injuries, e.g. transection of the anterior cruciate ligament, meniscectomy and destabilization of the medial meniscus (DMM). In these studies, it is important to accurately measure subchondral bone parameters, which are considered important targets for evaluating the disease progression and the efficacy of drugs and interventions.

The gold standard for measuring bone parameters in mouse models is micro-Computed Tomography (microCT) imaging (4). *Ex vivo* microCT is the predominant technique used to scan the mouse knee in OA studies (2, 5–10). However, in a cross-sectional design disease progression can only be evaluated by using multiple groups of mice sacrificed at different time points. *In vivo* microCT enables the longitudinal assessment of the joint of the same mouse over time, reducing the measurement variability and the number of animals required for the experiments (11), in line with the principles of the 3Rs (replacement, refinement and reduction of the usage of animals in research). However, to the authors’ knowledge, only three previous studies applied *in vivo* imaging for the characterization of subchondral bone in OA mice (12–14). This is probably because *in vivo* microCT is characterized by lower resolution and signal-to-noise ratio, due to the need of reducing scanning time and radiation exposure to the animal, which may impair the reliability and reproducibility of the measurements (15, 16). However, in the above-mentioned studies the accuracy in measuring the morphometric bone parameters was not reported for the applied scanning procedures, and very limited information about radiation effects were reported only in one of the studies [non-significant reduction in proximal tibia bone volume fraction (12)].

In order to increase the uptake of *in vivo* microCT imaging, reducing the group measurement variability by normalizing for baseline measurements, and decreasing the number of mice used in this research area, it is important to assess its accuracy in measuring subchondral bone morphological properties against the gold standard *ex vivo* microCT approach which allows to scan at higher resolution. The effect of image resolution in measuring subchondral bone parameters has been evaluated in a previous study for 10-week-old male C57BL/6 mice (N=5; no DMM surgery) (5). In particular, *ex vivo* images (5μm voxel size) were used as reference to assess the measurement accuracy in images acquired using an *in vivo* scanning procedure (10μm voxel size). Nevertheless, only a limited number of parameters (trabecular and cortical bone volume, subchondral plate thickness and trabecular BV/TV) were assessed in this study. Moreover, while good to weak correlations were found for some parameters of the subchondral bone measured with the two procedures (0.66<R^2^<0.88), surprisingly no correlation was found for trabecular bone volume in the medial and lateral compartments and for the bone volume fraction in the lateral compartment. It should be noted that this assessment depends dramatically on the quality of the images acquired *in vivo*, which depends on the used scanning parameters. In another study the accuracy of *in vivo*-microCT-based morphometric parameters measured in the L5 vertebra of C57BL/6N mice has been reported (17). The effect of the voxel size (from 6 to 30 μm) and segmentation method on trabecular morphometric parameters led to differences of up to 126% for measurements of trabecular thickness compared to high resolution images. Finally, in a previous study from our group, an optimal *in vivo* microCT scanning procedure was developed for imaging the whole mouse tibia, in order to minimize the effect of ionizing radiation on the bone remodelling (18) and maximizing the accuracy of the assessment of morphometric and densitometric parameters (19). Nevertheless, this assessment was performed only on the metaphyseal trabecular bone and it remains to be investigated what is the accuracy of *in vivo* microCT imaging to assess the tibia subchondral bone.

The aim of this study was to evaluate the ability of *in vivo* microCT imaging in measuring the microstructural properties of the mouse proximal tibia trabecular and cortical subchondral bone, with respect to *ex vivo* high-resolution imaging, in a DMM model of PTOA.

## 2 Materials and methods

### 2.1 Animals and intervention

Sixteen 14-weeks-old C57BL/6 (BL6) male mice were purchased from Charles River UK Ltd., Margate, UK. They were housed at the University of Sheffield’s Biological Services Unit with a twelve-hour light/dark cycle at 22°C and free access to food and water. All procedures were performed under a British Home Office project license and in compliance with the UK Animals (Scientific Procedures) Act 1986. This study was reviewed and approved by the local Research Ethics Committee of the University of Sheffield (Sheffield, UK). At 14-weeks-old DMM surgery was performed on the right knee joints for eight of the 16 mice. SHAM-operation (SHAM) on the right knee joint was performed on the remaining mice (n=8). At 26-weeks-old, mice were culled, and the legs were dissected from the body.

### 2.2 *In vivo* and *ex vivo* microCT imaging

In this study, *in vivo* and *ex vivo* microCT scans of the right knee for DMM and SHAM operated mice, were compared. The *ex vivo* microCT images were used as a gold standard to quantify the measurement uncertainties from the lower resolution *in vivo* microCT images.

A baseline scan of the right knee of each mouse before surgeries was performed at weeks 14 of age with *in vivo* microCT (VivaCT 80, Scanco Medical, Bruettisellen, Switzerland). The scanning parameters and the image processing procedure were optimized in previous studies to have a reproducible measurement yet minimizing the effect of the radiation dose on bone adaptation (18, 19). The following scanning parameters were used: 55 kVp, 145µA, 10.4 µm voxel size, 100 ms integration time, 32 mm field of view, 750 projections/180°, no frame averaging, 0.5 mm Al beam hardening filter. Afterwards, the right knee of each mouse was scanned with the same parameters at week 18, 22, and 26 of age. Each image was reconstructed using the software provided by the manufacturer using a polynomial beam hardening correction function based on the scans of a wedge phantom with 1200 mg HA/cc density, as recommended by (20).

At week 26 of age each mouse was culled and the right knee was imaged with *ex vivo* microCT (SkyScan 1172, Bruker, Belgium; 49 kV, 179 µA, 4.35 µm voxel size, 1180 ms exposure time, 180° rotation, 0.7° rotation step, frame averaging x2, 0.5 mm A1 filter) (19). Each *ex vivo* microCT image was reconstructed by using the software provided by the manufacturer (NRecon, Bruker; ring artifacts reduction factor 10, dynamic range 0–0.13) (19).

### 2.3 Image preprocessing and 3D morphometric analysis

**Figure 1:**
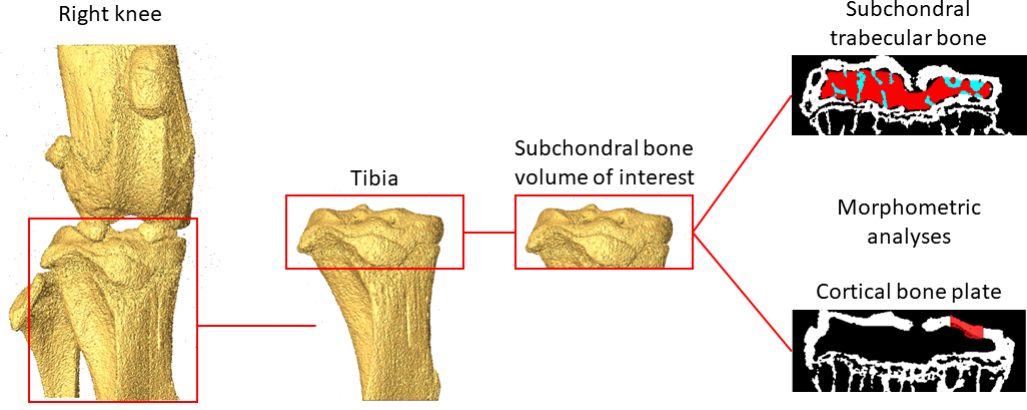
Example of the analysed regions of interest in the subchondral trabecular bone and cortical bone plate.

Scans of the right proximal tibia acquired at week 26 of age *ex vivo* and *in vivo* were compared in this study. Three-dimensional morphometric analyses were performed on *ex vivo* and *in vivo* microCT images of the proximal tibia after alignment. The femur, fibula and menisci were removed from each image (**Figure 1**) by applying a connectivity filter (connectivity rule = 6, bwlabeln function, MATLAB). Disconnections between adjacent bones were created manually where necessary. The *in vivo* microCT image of one tibia (age of 26 weeks) was randomly chosen and used as a reference. The reference tibia was rotated (Amira 6.3.0, FEI Visualization Sciences Group, France) in order to approximately align its longitudinal axis with the Z-axis of a global reference system. Rigid image registration was then applied to align the other images to the same reference system by using Normalized Mutual Information as optimization criterion and Lanczos interpolation for resampling (19, 21).

A Gaussian filtration (convolution kernel [3x3x3], standard deviation = 0.65, smooth3 function, MATLAB) was applied to reduce the high-frequency noise. Bone voxels were defined using a global threshold (MATLAB), which was calculated as the average of the grey levels corresponding to the bone and background peaks in each image histogram (19, 21). This approach was used to avoid operator-dependent selection of the threshold value and was found to be optimal from previous studies performed on the mouse tibia metaphysis [Supplementary material of (19)].

Binary datasets were imported in CTAn (Bruker, Belgium) for 3D morphometric analyses.

Trabecular bone regions of interest (ROIs) were identified by manually contouring the trabecular bone between the subchondral plate and growth plate in 2D cross-sections (**Figure 1**), from the *in vivo* microCT images every 5 slices. In order to match the analysed volumes, the same ROIs identified from the *in vivo* microCT images were used to analyze the morphometric properties of the subchondral trabecular bone in the *ex vivo* images. Two additional ROIs were generated for each image by dividing the trabecular ROI into medial and lateral compartments. Trabecular analyses were performed for the whole subchondral trabecular bone, for the medial compartment and for the lateral compartment. A despeckling filter was applied to remove the isolated bone regions with volume lower than 10 voxels. The following parameters were evaluated: trabecular bone volume fraction (BV/TV), trabecular thickness (Tb.Th), trabecular separation (Tb.Sp), trabecular number (Tb.N), bone surface density (BS/BV), degree of anisotropy (DA), structure model index (SMI) and connectivity density (Conn.D) (4).

For the cortical subchondral plate analysis, two ROIs were identified, one in the medial and one in the lateral portions of the tibia plateau, after removing the underlying trabecular bone (**Figure 1**). Each ROI, centered in each condyle, was identified with the same size (500 µm x 500 µm) and same spatial position for all images. Pores within the subchondral bone plate were removed by applying a closing operation in 2D (kernel = round, radius = 10 pixels). The subchondral bone cortical plate thickness (Ct.Th) was calculated for the medial (Ct.Th.Med.) and the lateral (Ct.Th.Lat.) ROIs (4).

### 2.4 Statistical analysis

Statistical analysis was performed in MATLAB (R2020a, MathWorks, USA). Paired T-tests (ttest function) were used to evaluate the differences between *in vivo* imaging resolution (10.4 µm voxel size) and *ex vivo* imaging resolution (4.35 µm voxel size). Differences were considered statistically significant when *p*-value was < 0.05. Linear regression analyses and Bland-Altman plots were used to assess the agreement between the morphometric properties measured from the *in vivo* or the *ex vivo* microCT images, for both pooled data (including both DMM and SHAM groups) or for separate groups. For each statistically significant relationship the coefficient of determination (R^2^) and the regression equation have been reported. For each parameter, normality of the differences between *ex vivo* and *in vivo* measurements was tested using the Lilliefors test (lillietest function, MATLAB).

The effect of DMM on the morphometric properties was evaluated both from *ex vivo* and *in vivo* microCT images. The differences between the DMM and SHAM mice were compared with two-tails T-tests (ttest2 function, MATLAB).

## 3 Results

**Figure 2:**
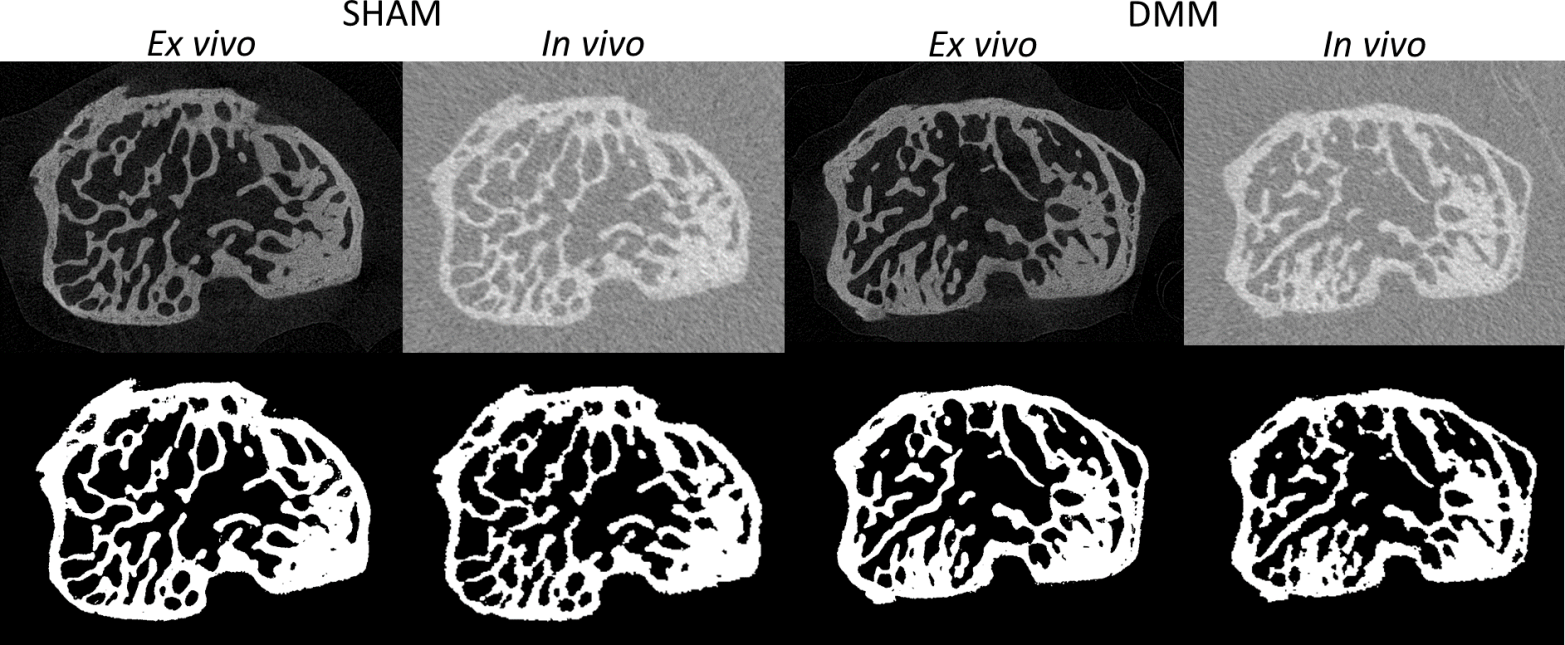
Examples of microCT cross-sections acquired *in vivo* and *ex vivo* (top), for a mouse in the SHAM group (left) and a mouse in the DMM group (right). Binary images after image segmentation are reported below each image.

**Figure 3:**
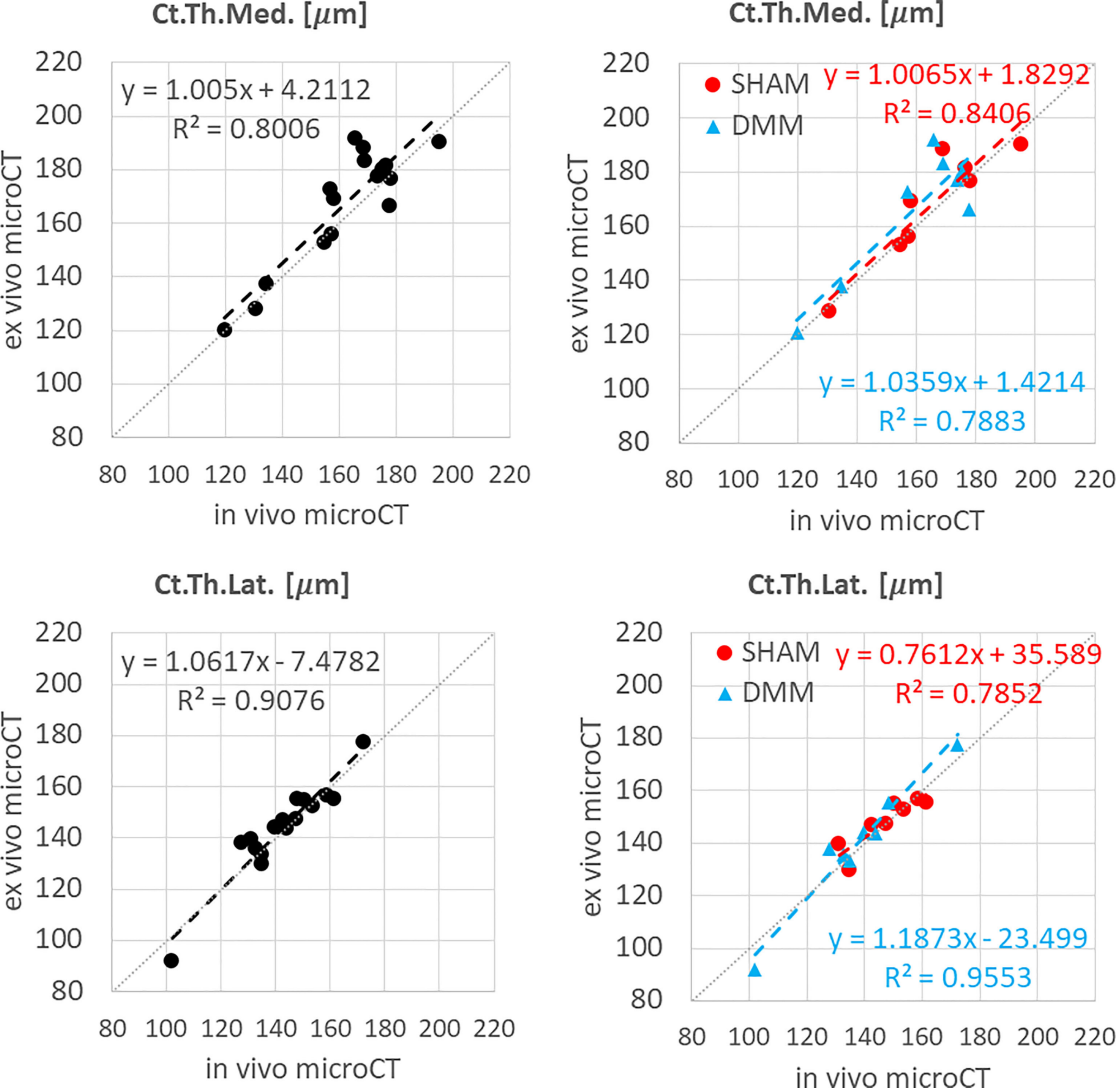
Regression analyses between *in vivo* and *ex vivo* measurements for cortical thickness (Ct.Th) at the medial and lateral side.

**Figure 4:**
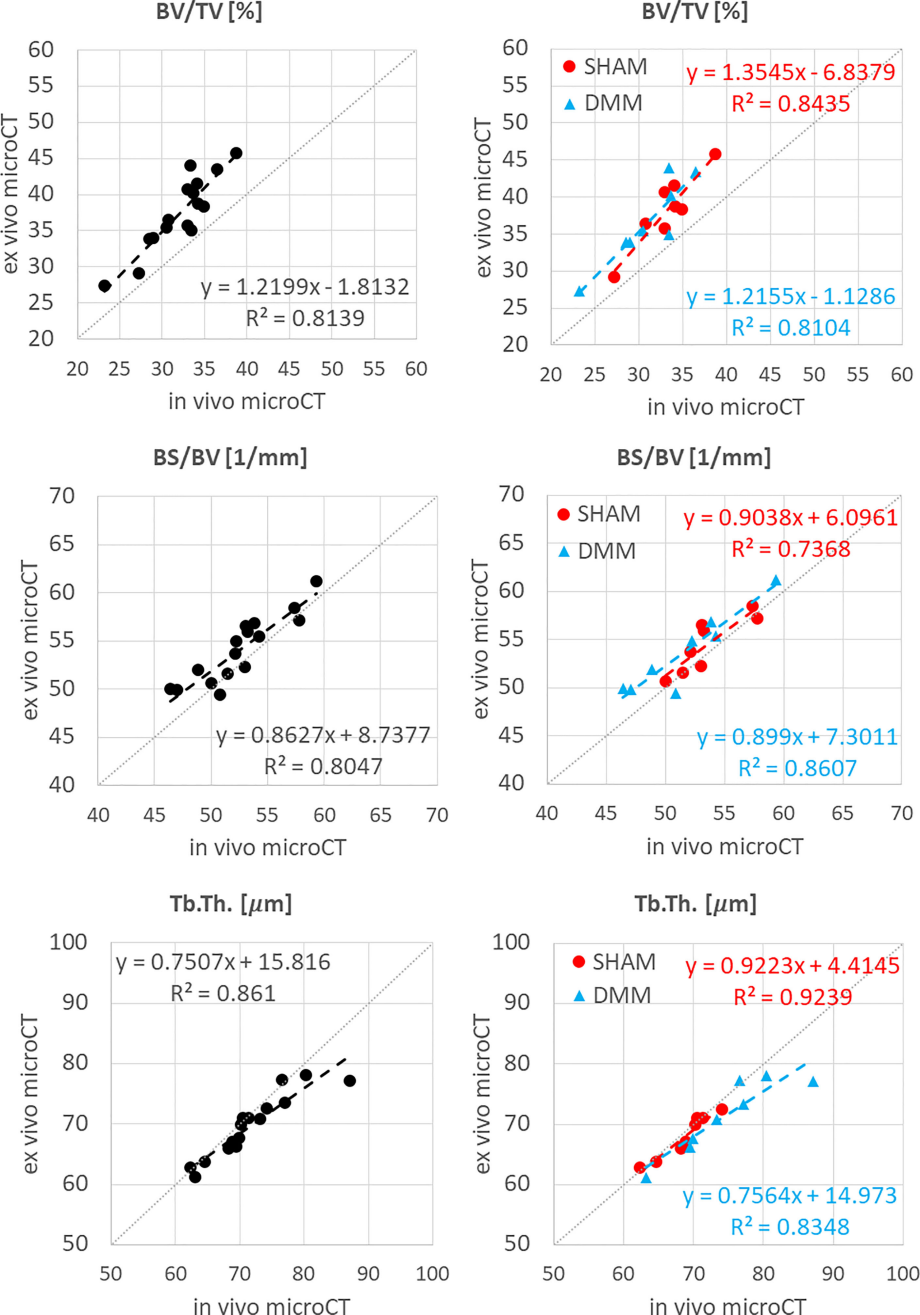
Regression analyses between *in vivo* and *ex vivo* measurements for bone volume fraction (BV/TV), bone surface fraction (BS/BV) and trabecular thickness (Tb.Th).

**Figure 5:**
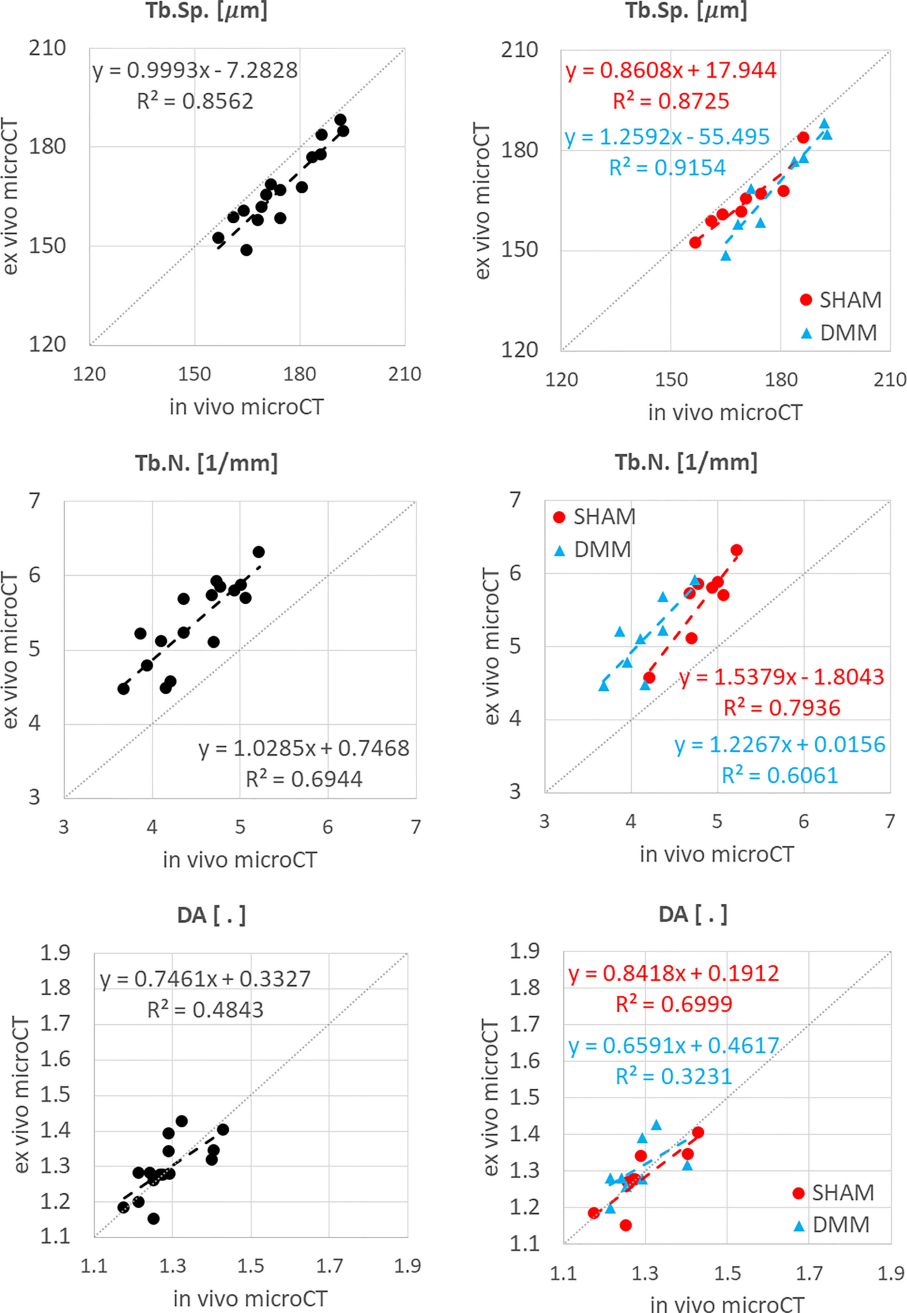
Regression analyses between *in vivo* and *ex vivo* measurements for trabecular separation (Tb.Sp), number (Tb.N) and degree of anisotropy (DA).

**Figure 6:**
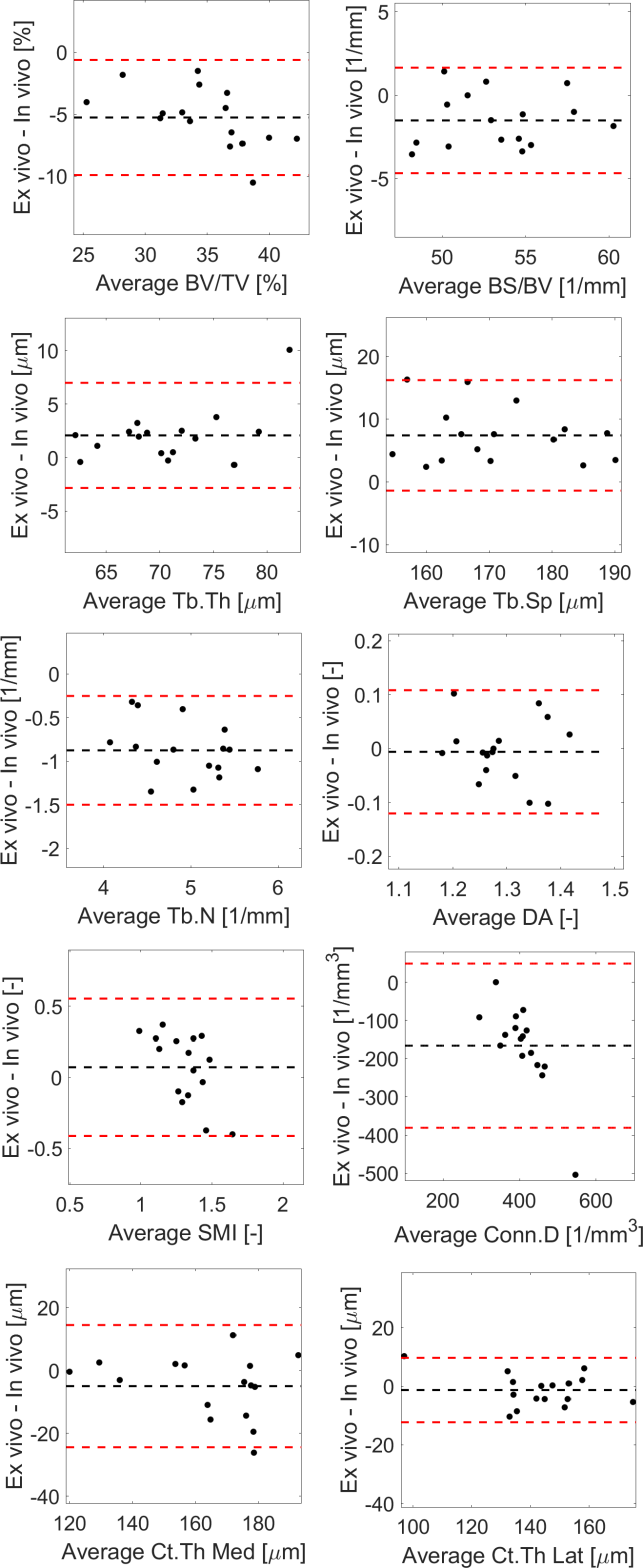
Bland-Altman plots between *in vivo* and *ex vivo* measurements for trabecular bone volume fraction (BV/TV), bone surface fraction (BS/BV), trabecular thickness (Tb.Th), separation (Tb.Sp), number (Tb.N) and degree of anisotropy (DA), structure model index (SMI), connectivity density (Conn.D), and cortical thickness (Ct.Th) in the medial or lateral side.

Examples of microCT images acquired *in vivo* and *ex vivo* are reported in **Figure 2** for both SHAM and DMM groups. The descriptive statistics for the different microstructural properties calculated from the *in vivo* and *ex vivo* microCT scans are reported in **Table 1**. Regression analyses for each morphometric parameter are reported in **Figures 3**–**5**, while Bland-Altman plots are shown in **Figure 6**.

**Table 1:** Morphometric parameters measured with *in vivo* and *ex vivo* microCT for the pooled (ALL) data and for each group (SHAM or DMM).

	ALL				SHAM				DMM			
	*ex vivo*	*in vivo*	diff	%diff	*ex vivo*	*in vivo*	diff	%diff	*ex vivo*	*in vivo*	diff	%diff
**BV/TV [%]**	37.4 ± 5.1	32.2 ± 3.8	-5.3 ± 2.4	-13.7 ± 5.1*	38.2 ± 4.9	33.3 ± 3.3	-5.0 ± 2.3	-12.6 ± 4.8*	36.6 ± 5.6	31.1 ± 4.1	-5.6 ± 2.6	-14.9 ± 5.3*
**BS/BV [1/mm]**	54.1 ± 3.5	52.6 ± 3.6	-1.5 ± 1.6	-2.8 ± 3.1*	54.5 ± 2.9	53.6 ± 2.7	-0.9 ± 1.5	-1.7 ± 2.7	53.7 ± 4.1	51.6 ± 4.3	-2.1 ± 1.6	-3.9 ± 3.2*
**Tb.Th. [µm]**	70 ± 5	72 ± 6	2 ± 3	2.9 ± 3.3*	68 ± 4	69 ± 4	1 ± 1	1.4 ± 1.6*	71 ± 6	75 ± 7	3 ± 3	4.5 ± 3.9*
**Tb.Sp.[µm]**	167 ± 12	175 ± 11	7 ± 4	4.5 ± 2.9*	165 ± 9	170 ± 10	6 ± 4	3.5 ± 2.1*	170 ± 14	179 ± 11	9 ± 5	5.5 ± 3.4*
**Tb.N.[1/mm]**	5.4 ± 0.6	4.5 ± 0.5	-0.9 ± 0.3	-16.1 ± 5.4*	5.6 ± 0.5	4.8 ± 0.3	-0.8 ± 0.3	-13.8 ± 4.4*	5.1 ± 0.5	4.2 ± 0.3	-1.0 ± 0.3	-18.4 ± 5.5*
**DA [.]**	1.3 ± 0.1	1.3 ± 0.1	0.0 ± 0.1	-0.3 ± 4.5	1.3 ± 0.1	1.3 ± 0.1	0.0 ± 0.0	1.1 ± 3.9	1.3 ± 0.1	1.3 ± 0.1	0.0 ± 0.1	-1.8 ± 4.7
**SMI [.]**	1.3 ± 0.3	1.4 ± 0.1	0.1 ± 0.2	9.0 ± 20.0	1.2 ± 0.3	1.3 ± 0.1	0.1 ± 0.3	10.0 ± 24.3	1.4 ± 0.2	1.4 ± 0.1	0.1 ± 0.2	7.9 ± 16.3
**Conn.D. [1/mm^3^]**	490 ± 108	324 ± 34	-166 ± 110	-31.5 ± 13.5*	479 ± 76	347 ± 14	-132 ± 77	-25.9 ± 13.3*	502 ± 138	302 ± 33	-200 ± 132	-37.1 ± 12.0*
**Ct.Th.Med.[µm]**	167 ± 22	162 ± 20	-5 ± 10	-2.7 ± 5.5	168 ± 21	165 ± 19	-3 ± 8	-1.5 ± 4.7	166 ± 25	159 ± 21	-7 ± 11	-3.9 ± 6.2
**Ct.Th.Lat. [µm]**	144 ± 18	143 ± 16	-1 ± 6	-0.6 ± 4.5	148 ± 9	148 ± 11	0 ± 5	-0.2 ± 3.5	140 ± 24	138 ± 20	-2 ± 6	-1.0 ± 5.6

Differences are reported as average ± SD. *significant differences (p<0.05). In bold values that were found to be significantly different.

*In vivo* and *ex vivo* microCT measurements of cortical thickness were strongly correlated at both medial (R^2^ = 0.80) and lateral (R^2^ = 0.91) sides (**Figure 3**), with no significant differences between the two imaging modalities (**Table 1**).

The total trabecular BV/TV and the BS/TV calculated with *in vivo* or *ex vivo* microCT imaging were strongly correlated for pooled data (R^2 ^= 0.81 for BV/TV and R^2^ = 0.80 for BS/TV) and for each group (SHAM group: R^2^ = 0.84 for BV/TV and R^2^ = 0.74 for BS/TV; DMM: R^2^ = 0.81 for BV/TV and R^2^ = 0.86 for BS/TV) (**Figure 4**). Nevertheless, the BV/TV was systematically underestimated (mean difference of -13.7% ± 5.1%, p<0.001) by the *in vivo* microCT analyses. Underestimation was similar for the SHAM and DMM groups (**Table1**).

Similarly, Tb.Th. and Tb.Sp. calculated in the whole trabecular ROI with *in vivo* or *ex vivo* microCT imaging were strongly correlated for pooled data (R^2^ = 0.86 for both Tb.Th. and Tb.Sp.) and for each group (SHAM group: R^2^ = 0.92 for Tb.Th. and R^2^ = 0.87 for Tb.Sp.; DMM group: R^2^ = 0.83 for Tb.Th. and R^2^ = 0.92 for Tb.Sp.) (**Figure 4**, **5**). Nevertheless, the Tb.Th. was slightly overestimated (mean difference of 2.9% ± 3.3%, p=0.005) by the *in vivo* microCT analyses, with a little higher difference for the DMM group (4.5% ± 3.9%, p=0.021) than for the SHAM group (1.4% ± 1.6%, p=0.039) (**Table 1**). Similarly, the Tb.Sp. was overestimated (mean difference of 4.5% ± 2.9%, p<0.001) by the *in vivo* microCT analyses, with higher differences for the DMM group (5.5% ± 3.4%, p=0.001), compared to the SHAM group (3.5% ± 2.1%, p=0.002) (**Table 1**).

Significant but worse correlations were found for Tb.N calculated in the whole trabecular ROI (R^2^ = 0.69 for pooled data, R^2^ = 0.79 for SHAM, R^2^ = 0.61 for DMM) (**Figure 5**), which was systematically underestimated by the *in vivo* microCT images (ALL: -16.1% ± 5.4%; SHAM: -13.8% ± 4.4%; DMM: -18.4% ± 5.5%, all with p<0.001) (**Table 1**).

No significant differences were found between *in vivo* and *ex vivo* measurements of DA and SMI calculated in the whole subchondral trabecular bone. A weak correlation between *in vivo* and *ex vivo* microCT data was found for DA for pool data (R^2^ = 0.48 for pooled data), with stronger correlations for the individual groups (R^2^ = 0.79 for SHAM, R^2^ = 0.61 for DMM) (**Figure 5**). Conn.D was systematically underestimated by *in vivo* microCT imaging (differences between -37.1% and -25.9%, **Table 1**). No significant correlation was found between *in vivo* and *ex vivo* microCT measurements for SMI and Conn.D (p>0.05).

Most results in the Bland-Altman plots lied within ±2 SD of the mean difference, indicating good agreement between the two methods (22). Differences between the two methods were normally distributed for all parameters except Tb.Th (however distribution was normal when removing one outlier, **Figure 6**). A bias was found for the two parameters that were not significantly linearly correlated (Conn.D and SMI).

**Figure A1:**
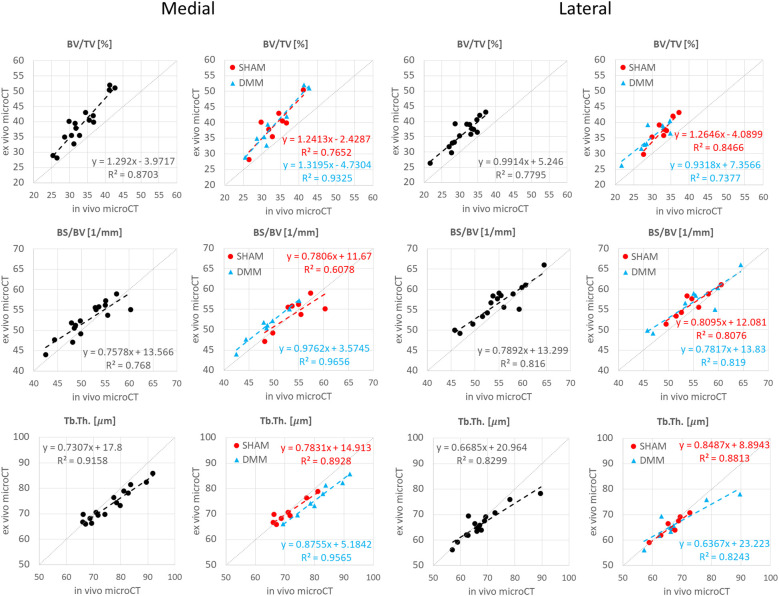
Regression analyses between in vivo and ex vivo measurements for bone volume fraction (BV/TV), bone surface fraction (BS/BV) and trabecular thickness (Tb.Th) in the medial and lateral compartments.

**Figure A2:**
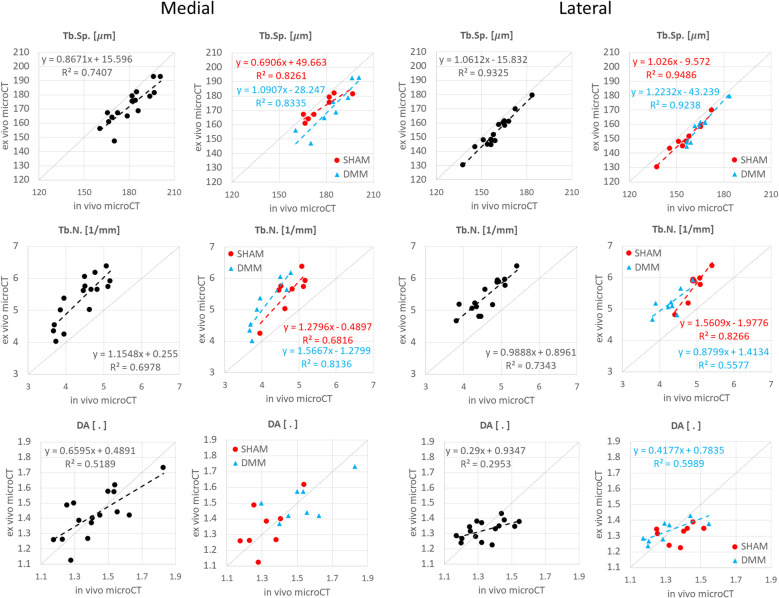
Regression analyses between in vivo and ex vivo measurements for trabecular separation (Tb.Sp), number (Tb.N) and degree of anisotropy (DA) in the medial and lateral compartments.

The descriptive statistics for the trabecular parameters calculated for the sub-ROIs in the medial or lateral compartments are reported in **Tables 2**, **3** (detailed regression analyses are reported in **Appendix A1**, **Figure A1** and **Figure A2**). Results were consistent with those found for the whole trabecular ROI, with *in vivo* microCT imaging underestimating BV/TV (-12% to -15%) and Tb.N (-14% to -20%), and overestimating Tb.Sp (3% to 7%). BS/BV was slightly underestimated only in the lateral region (-3.1%). Tb.Th was slightly overestimated only in the medial compartment (+3.6%).

**Table 2:** Trabecular parameters measured in the medial compartment with *in vivo* and *ex vivo* microCT for the pooled (ALL) data and for each group (SHAM or DMM).

	ALL				SHAM				DMM			
	*ex vivo*	*in vivo*	diff	%diff	*ex vivo*	*in vivo*	diff	%diff	*ex vivo*	*in vivo*	diff	%diff
**BV/TV[%]**	39.4 ± 7.2	33.6 ± 5.2	-5.8 ± 3.0	-14.2 ± 6.0*	39.4 ± 6.3	33.7 ± 4.5	-5.7 ± 3.3	-13.9 ± 7.0*	39.5 ± 8.4	33.5 ± 6.1	-6.0 ± 2.9	-14.6 ± 5.3*
**BS/BV [1/mm]**	52.6 ± 4.1	51.5 ± 4.8	-1.1 ± 2.3	-2.1 ± 4.3	53.9 ± 3.9	54.1 ± 3.9	0.2 ± 2.6	0.5 ± 4.7	51.2 ± 4.1	48.8 ± 4.1	-2.4 ± 0.8	-4.7 ± 1.5*
**Tb.Th. [µm]**	73.5 ± 6.3	76.3 ± 8.2	2.7 ± 2.9	3.6 ± 3.7*	70.7 ± 4.6	71.2 ± 5.5	0.5 ± 1.9	**0.7 ± 2.7**	76.4 ± 6.7	81.3 ± 7.5	4.9 ± 1.7	6.4 ± 2.0*
**Tb.Sp. [µm]**	172.2 ± 12.5	180.6 ± 12.4	8.4 ± 6.6	5.0 ± 4.2*	172.1 ± 8.3	177.3 ± 10.9	5.2 ± 4.8	3.0 ± 2.7*	172.2 ± 16.3	183.8 ± 13.6	11.6 ± 6.8	7.0 ± 4.6*
**Tb.N. [1/mm]**	5.3 ± 0.7	4.4 ± 0.5	-0.9 ± 0.4	-17.1 ± 6.5*	5.5 ± 0.6	4.7 ± 0.4	-0.8 ± 0.4	-14.5 ± 5.8*	5.2 ± 0.8	4.1 ± 0.5	-1.0 ± 0.4	-19.7 ± 6.4*
**DA [.]**	1.4 ± 0.2	1.4 ± 0.2	0.0 ± 0.1	0.0 ± 8.7	1.4 ± 0.2	1.3 ± 0.1	0.0 ± 0.1	-1.4 ± 9.2	1.5 ± 0.1	1.5 ± 0.2	0.0 ± 0.1	1.3 ± 8.5
**SMI [.]**	1.4 ± 0.4	1.5 ± 0.2	0.1 ± 0.3	15.1 ± 23.9	1.3 ± 0.4	1.5 ± 0.2	0.1 ± 0.3	15.9 ± 27.7	1.5 ± 0.4	1.6 ± 0.1	0.1 ± 0.3	14.4 ± 21.2

Differences are reported as average ± SD. *significant differences (p<0.05). In bold values that were found to be significantly different.

**Table 3:** Trabecular parameters measured in the lateral compartment with *in vivo* and *ex vivo* microCT for the pooled (ALL) data and for each group (SHAM or DMM).

	ALL				SHAM				DMM			
	*ex vivo*	*in vivo*	diff	%diff	*ex vivo*	*in vivo*	diff	%diff	*ex vivo*	*in vivo*	diff	%diff
**BV/TV [%]**	36.2 ± 4.6	31.2 ± 4.1	-5.0 ± 2.1	-13.6 ± 5.3*	37.5 ± 4.2	32.9 ± 3.0	-4.6 ± 1.8	-12.1 ± 4.1*	34.9 ± 4.8	29.6 ± 4.5	-5.3 ± 2.5	-15.2 ± 6.1*
**BS/BV [1/mm]**	56.6 ± 4.4	54.8 ± 5.0	-1.7 ± 2.2	-3.1 ± 3.9*	56.3 ± 3.2	54.6 ± 3.6	-1.7 ± 1.6	-3.0 ± 2.7*	56.8 ± 5.5	55.0 ± 6.4	-1.8 ± 2.7	**-3.3 ± 5.0**
**Tb.Th [µm]**	66.2 ± 5.7	67.6 ± 7.7	1.5 ± 3.5	2.1 ± 4.7	65.4 ± 3.8	66.6 ± 4.2	1.2 ± 1.5	1.8 ± 2.3	67.0 ± 7.3	68.7 ± 10.4	1.7 ± 4.8	2.3 ± 6.5
**Tb.Sp [µm]**	153.5 ± 11.8	159.6 ± 10.7	6.1 ± 3.1	4.0 ± 2.2*	149.4 ± 11.5	154.9 ± 10.9	5.5 ± 2.6	3.8 ± 1.9*	157.7 ± 11.3	164.2 ± 8.9	6.6 ± 3.7	4.3 ± 2.6*
**Tb.N [1/mm]**	5.5 ± 0.5	4.6 ± 0.4	-0.8 ± 0.3	-15.3 ± 4.6*	5.7 ± 0.5	4.9 ± 0.3	-0.8 ± 0.3	-13.6 ± 3.8*	5.2 ± 0.4	4.3 ± 0.4	-0.9 ± 0.3	-17.1 ± 5.0*
**DA [.]**	1.3 ± 0.1	1.3 ± 0.1	0.0 ± 0.1	1.4 ± 7.3	1.3 ± 0.1	1.4 ± 0.1	0.1 ± 0.1	4.5 ± 7.1	1.3 ± 0.1	1.3 ± 0.1	0.0 ± 0.1	-1.7 ± 6.4
**SMI [.]**	1.3 ± 0.2	1.3 ± 0.1	0.0 ± 0.2	4.8 ± 16.8	1.2 ± 0.3	1.2 ± 0.1	0.0 ± 0.2	5.5 ± 20.7	1.4 ± 0.1	1.4 ± 0.1	0.0 ± 0.2	4.2 ± 13.2

Differences are reported as average ± SD. *significant differences (p<0.05). In bold values that were found to be significantly different.

**Figure 7:**
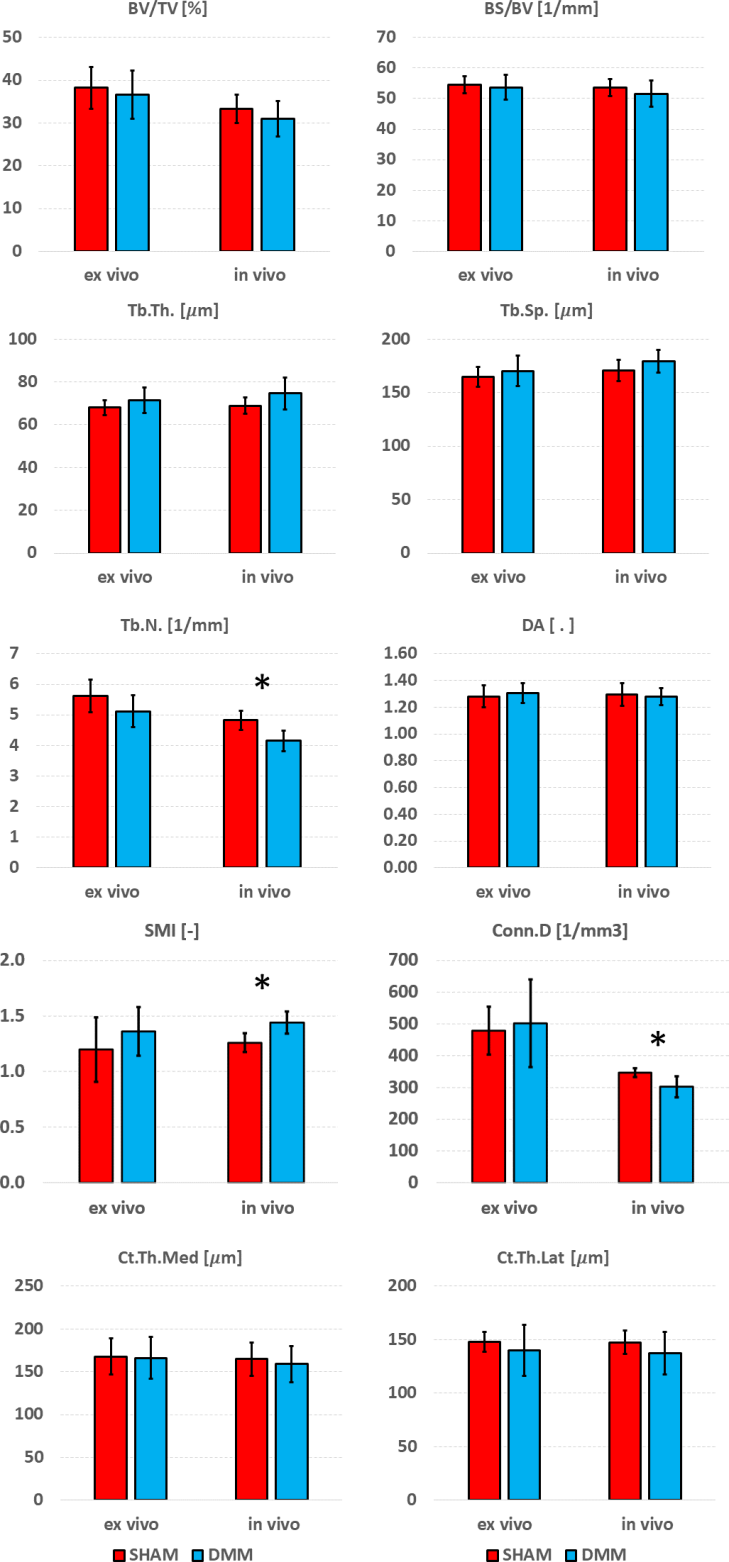
Effect of DMM surgery measured with *in vivo* and *ex vivo* microCT. Trabecular bone volume fraction (BV/TV), bone surface fraction (BS/BV), trabecular thickness (Tb.Th), trabecular separation (Tb.Sp), trabecular number (Tb.N), degree of anisotropy (DA), structure model index (SMI) and connectivity density (Conn.D), and cortical thickness (Ct.Th) at medial and lateral side. *significant differences (p<0.05).

**Figure 8:**
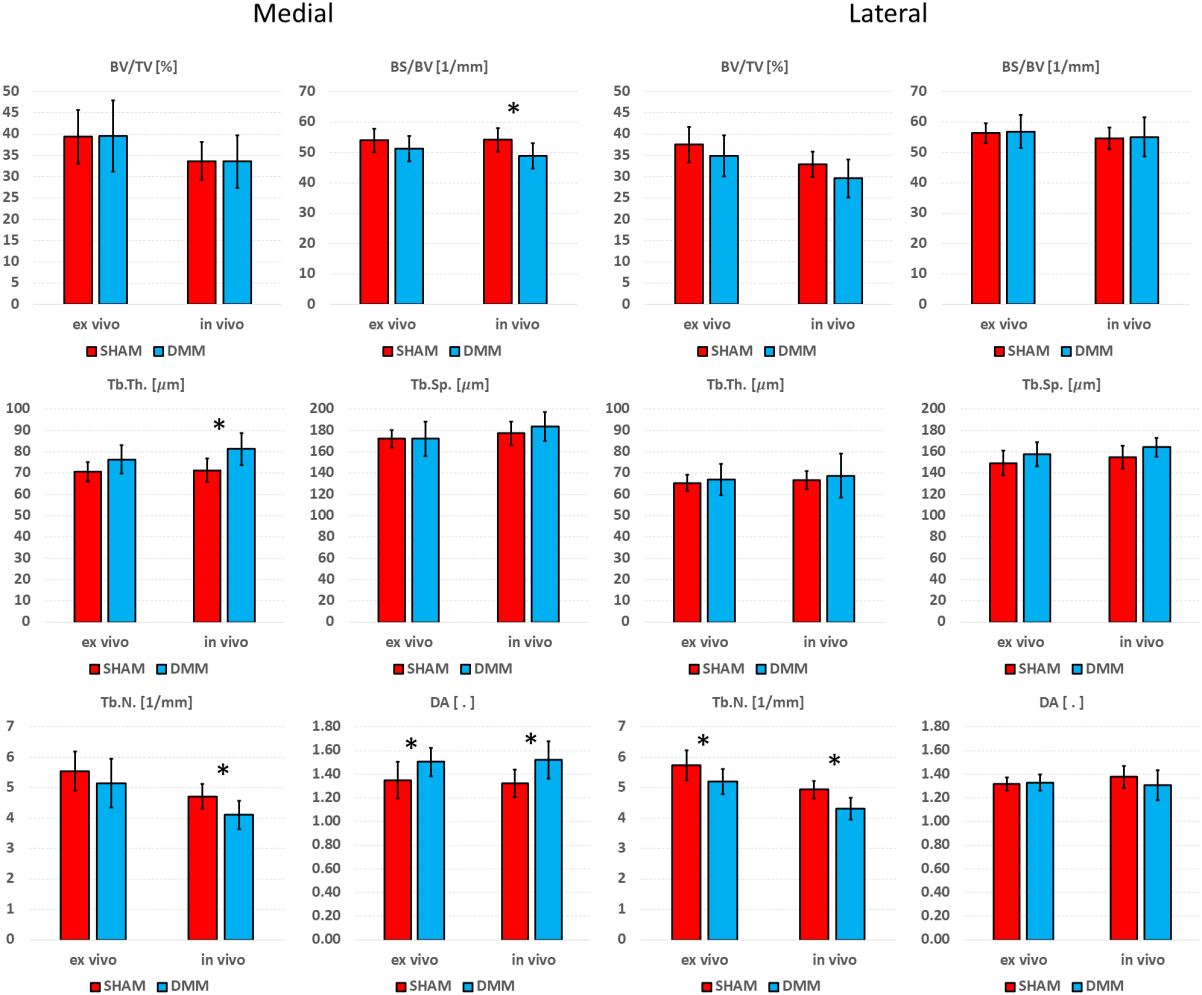
Effect of DMM surgery measured with in *in vivo* and *ex vivo* microCT in the medial and lateral trabecular compartments. Trabecular bone volume fraction (BV/TV), bone surface fraction (BS/BV), trabecular thickness (Tb.Th), trabecular separation (Tb.Sp), trabecular number (Tb.N), degree of anisotropy (DA). *significant differences (p<0.05).

Differences between SHAM and DMM groups when evaluated from *in vivo* and *ex vivo* microCT images are reported in **Figures 7** (analysis in the whole trabecular ROI) and **Figure 8** (analyses in the medial or lateral compartments). No significant differences were found in trabecular or cortical parameters measured with *ex vivo* microCT between the two groups. Tb.N, SMI and Conn.D measured with *in vivo* microCT were significantly different between SHAM and DMM groups. Differences were similar to those measured *ex vivo* for two of the measurements (-9.0% difference in Tb.N with *ex vivo* vs -13.9% with *in vivo*, +13.6% difference in SMI with *ex vivo* vs +14.5% with *in vivo*). A larger difference was found for Conn.D (+4.8% with *ex vivo* vs -12.8% with *in vivo*).

In the medial compartment, a significant difference in DA between SHAM and DMM groups was found with both *ex vivo* (+11.4%) and *in vivo* (+15.1%) microCT imaging. Differences in BS/BV (-5.0% *ex vivo* vs -9.9% *in vivo*), Tb.Th (+8.0% vs +14.1%) and Tb.N (-7.1% vs -13.0%) were only significant when measured *in vivo*. In the lateral compartment, a significant decrease in Tb.N was found for DMM mice both *ex vivo* (-9.1%) and *in vivo* (-12.7%).

## 4 Discussion

The goal of this study was to quantify the accuracy of *in vivo* microCT imaging to measure trabecular and cortical morphometric parameters in the subchondral bone of the proximal mouse tibia. The results show that *in vivo* and *ex vivo* measurements were highly correlated for most trabecular parameters, even though *in vivo* microCT over- or under-estimated some of them. Cortical plate thickness was accurately measured with *in vivo* imaging.

Lower resolution imaging led to significant underestimation of bone volume fraction (-13.7%), bone surface density (-2.8%) and trabecular number (-16.1%), whereas trabecular thickness (+2.9%) and separation (+4.5%) were significantly overestimated. No significant differences between degree of anisotropy measured *in vivo* and *ex vivo* were found. The underestimation of BV/TV and Tb.N were likely due to the fact that smaller trabeculae or thin connections are not visible at lower resolution, which also leads to underestimation of Conn.D and overestimation of Tb.Sp. On the other hand, the overestimation of trabecular thickness could be due to the partial volume effect linked to the larger voxel size (10.4 µm *in vivo* vs 4.35 µm *ex vivo*). Nevertheless, the high correlation between *in vivo* and *ex vivo* measurements for most microstructural parameters indicates that variations are adequately captured by *in vivo* imaging, which therefore can be used to measure morphometric parameters over time, especially considering that in a longitudinal design systematic over- or under-estimations are compensated by measuring changes from baseline. Nevertheless, Conn.D and SMI values calculated with *in vivo* or *ex vivo* microCT were not correlated, showing that these two parameters should not be used when assessing the morphology of the subchondral trabecular bone from *in vivo* microCT images.

The results obtained in this study for some of the parameters are in contrast with those obtained for the vertebral and distal femur trabecular bone, for which it has been reported that increasing voxel size led to overestimation of bone volume fraction and trabecular thickness (17, 23). Nevertheless, these measurements depend from the threshold value used for segmentation (17). In this study thresholds were specimen-specific and calculated automatically, thus assuring measurement repeatability. Additionally, the voxel size of reference images (6 µm in (17), 8 µm in (23), 4.35 µm in this study) used as gold standard high resolution images plays a role in the measured errors.

Scanning method minimally affected cortical thickness measurements, which was consistent with previous studies (17, 19) and likely due to the simplified structure of cortical plate and higher ratio between thickness and voxel size.

Significant differences between SHAM and DMM groups were found with *in vivo* images for trabecular number (-13.9% in DMM) and structure model index (+14.5% in DMM), even though the measured differences were consistent with those obtained *ex vivo* which however were not statistically significant (-9.0% and +13.7%, respectively). When measuring trabecular parameters separately in the medial and lateral compartments, a significant increase in DA was observed in the DMM group at the medial side (+11.4% *ex vivo* and +15.1% *in vivo*) and a significant decrease in trabecular number at the lateral side (-9.1% vs -12.7%). BS/BV and Tb.N were consistently lower in the DMM group at the medial side, while Tb.Th was consistently higher, nevertheless these differences were only significant when measured *in vivo*. While it is surprising that most significant differences were found from *in vivo* microCT measurements, this may be due to the relatively low sample size considered in this study. Nevertheless, when the DMM model has been assessed in the literature, most differences occurred on the cartilage and on the osteophyte formation and volume. Little differences were found for some of the microstructural parameters. For example, using an approach similar to the *in vivo* microCT imaging used in this study (~10µm voxel size) on SHAM-operated and DMM mice (4 months old, 2 months after DMM) Huang et al. (24) did not find any differences in cortical plate properties but found differences in osteophyte volume and density. In another study using a microCT imaging similar to the *ex vivo* approach used in this study (~5µm voxel size) Das Neves Borges found differences at 12 weeks post DMM in the medial subchondral plate, and in the lateral trabecular TV, but no other morphometric parameters were studied. However, in that study the contralateral not-operated bone was used as control, which may as well be affected by the change in gait due to the surgery on the contralateral leg, making it unclear to isolate the effect of the DMM. Therefore, more research is required to assess early effects of DMM on the local microstructural properties of the trabecular subchondral bone.

A limitation of this study is that a simple global thresholding was used for trabecular bone segmentation, while *in vivo* measurements may be improved with more advanced segmentation methods (25, 26). Even though only one mouse strain was included in this analysis and results may vary for different strains, nevertheless C57BL/6 mice are most commonly used for DMM studies (2, 5, 8, 9, 27). Lastly, the *in vivo* scanning procedure used in this study was optimized in previous work aiming to minimize the effects of the ionizing radiation on bone adaptation over time at different time points (19). In fact, the image quality from the *in vivo* scans could be increased by increasing the scanning time (and therefore the radiation), which may be acceptable in *in vivo* studies where the animals are scanned only twice after several weeks. Nevertheless, the effect of the radiation on the bone adaptation in the subchondral bone should be quantified.

In conclusion, *in vivo* microCT was able to measure cortical morphometric parameters with excellent accuracy and most trabecular parameters with very good accuracy in the subchondral bone of the mouse tibia. *In vivo* imaging can be applied to study disease (e.g. OA) onset and progression, as well as the effects of treatments over time, providing important longitudinal information. *Ex vivo* scanning is recommended at the end of the study for more accurate microstructural characterization and confirm the *in vivo* results at that time point.

## Data availability statement

The original contributions presented in the study are included in the article/supplementary material. Further inquiries can be directed to the corresponding author.

## Author contributions

Conceptualisation: SO, HI, IB, ED; Data curation: SO, ED; Formal analysis: SO, EM, ED; Funding acquisition: IB, ED; Investigation: SO, EM, ZC, AR, BR, HI; Methodology: SO, EM, ZC, AR, BR, HI; Project administration: SO, ED; Resources: IB, ED; Software: SO, EM; Supervision: IB, HI, ED; Visualisation: SO, ED; Writing – Original Draft Preparation: SO; Writing – Review & Editing: ALL. All authors contributed to the article and approved the submitted version.
